# Adolescent Trauma During the COVID Pandemic: Just Like Adults, Children, or Someone Else?

**DOI:** 10.1177/00031348221101475

**Published:** 2022-10

**Authors:** Perisa Ruhi-Williams, Eric O. Yeates, Areg Grigorian, Morgan Schellenberg, Natthida Owattanapanich, Galinos Barmparas, Daniel Margulies, Catherine Juillard, Kent Garber, Henry Cryer, Areti Tillou, Sigrid Burruss, Liz Penaloza-Villalobos, Ann Lin, Ryan Arthur Figueras, Raul Coimbra, Megan Brenner, Todd Costantini, Jarrett Santorelli, Terry Curry, Diane Wintz, Walter L. Biffl, Kathryn B. Schaffer, Thomas K. Duncan, Casey Barbaro, Graal Diaz, Arianne Johnson, Justine Chinn, Ariana Naaseh, Amanda Leung, Christina Grabar, Jeffry Nahmias

**Affiliations:** 1 Department of Surgery, 21769 University of California, Irvine (UCI), Orange, CA, USA; 2 Department of Surgery, 23336 University of Southern California (USC), Los Angeles, CA, USA; 3 Department of Surgery, 22494 Cedars-Sinai Medical Center, Los Angeles, CA, USA; 4 Department of Surgery, 8783 University of California, Los Angeles (UCLA), Los Angeles, CA, USA; 5 Department of Surgery, 4608 Loma Linda University, Loma Linda, CA, USA; 6 472028 Riverside University Health System Medical CenterUniversity, Moreno Valley, CA, USA; 7 University of California, Riverside/Riverside University Health System Department of Surgery, Moreno Valley, CA, USA; 8 Department of Surgery, 8784 University of California, San Diego (UCSD), San Diego, CA, USA; 9 Department of Surgery, 21380 Sharp Memorial Hospital, San Diego, CA, USA; 10 Trauma Department, 24146 Scripps Memorial Hospital La Jolla, La Jolla, CA, USA; 11 Department of Surgery, 25352 Ventura County Medical Center, Ventura, CA, USA; 12 Cottage Health Research Institute, 22854 Santa Barbara Cottage Hospital, Santa Barbara, CA, USA

**Keywords:** COVID-19, adolescent, trauma, pandemic

## Abstract

COVID-19 stay-at-home (SAH) orders were impactful on adolescence, when social interactions affect development. This has the potential to change adolescent trauma. A post-hoc multicenter retrospective analysis of adolescent (13-17 years-old) trauma patients (ATPs) at 11 trauma centers was performed. Patients were divided into 3 groups based on injury date: historical control (CONTROL:3/19/2019-6/30/2019, before SAH (PRE:1/1/2020-3/18/2020), and after SAH (POST:3/19/2020-6/30/2020). The POST group was compared to both PRE and CONTROL groups in separate analyses**.** 726 ATPs were identified across the 3 time periods. POST had a similar penetrating trauma rate compared to both PRE (15.8% vs 13.8%, *P* = .56) and CONTROL (15.8% vs 14.5%, *P* = .69). POST also had a similar rate of suicide attempts compared to both PRE (1.2% vs 1.5%, *P* = .83) and CONTROL (1.2% vs 2.1%, *P* = .43). However, POST had a higher rate of drug positivity compared to CONTROL (28.6% vs 20.6%, *P* = .032), but was similar in all other comparisons of alcohol and drugs to PRE and POST periods (all *P* > .05). Hence ATPs were affected differently than adults and children, as they had a similar rate of penetrating trauma, suicide attempts, and alcohol positivity after SAH orders. However, they had increased drug positivity compared to the CONTROL, but not PRE group.

## Key Takeaways

- COVID-19 stay-at-home orders had effects on adolescent trauma patients which differed than the effects on adults and pediatric trauma patients.
- Adolescent trauma patients had a similar rate of penetrating trauma, suicide attempts, and alcohol positivity after stay-at-home orders.
- Adolescent trauma patients had increased drug positivity after stay-at-home orders compared to a historical control population.

## Introduction

Coronavirus disease 19 (COVID-19), caused by severe acute respiratory syndrome coronavirus 2 (SARS-CoV-2), has been particularly devastating with nearly 5.5 million confirmed deaths worldwide.^1^ To curtail transmission of the virus, many regions established stay-at-home (SAH) orders. These SAH orders, although necessary to reduce viral spread, have also caused social isolation, psychological distress,^2^ and increased substance abuse.^3^

New studies have shown multiple effects of COVID-19 SAH orders on adult trauma populations including increased amphetamine, 3,4-methylenedioxy-methamphetamine (MDMA), and tetrahydrocannabinol (THC) positivity.^4^ Firearm deaths and penetrating trauma rates were also found to be increased both in the Southern California region and other parts of the United States.^5,6^ However, some studies including only pediatric trauma patients have shown no difference in penetrating trauma rates after SAH orders, suggesting that different age groups have been affected differently.^7^

The adolescent population faces a unique set of stressors. For example, the emotional effects of COVID-19 have been particularly impactful on adolescents who are at an age where social interactions are paramount to their development.^8^ In the United States, intentional self-harm (suicide) is the second leading cause of death among those aged 10-19 years^9^ and suicidal ideation in adolescents has reportedly risen during the COVID-19 pandemic.^10^ Adolescence is also a time of increased risk-taking^11^ and substance use is often initiated during these years of development^12^ and could be accentuated by pandemic related stressors. These predilections have potential to change the makeup of the adolescent trauma population during SAH orders, an area that has not yet been explored. Therefore, this study aimed to examine changes in adolescent trauma during the COVID-19 pandemic. We hypothesized an increased rate of penetrating traumas, suicide, and drug and alcohol positivity.

## Methods

This study was approved by the Institutional Review Board of the University of California, Irvine, as well as all other participating institutions, and was deemed exempt from the need for consent. A post-hoc multicenter retrospective analysis of adolescent (13-17 years-old) trauma patients presenting to 11 American College of Surgeons (ACS) Level-I and Level-II trauma centers in Southern California was performed. These 11 centers are comprised of both private and academic hospitals that span seven counties. No adolescent trauma patients were excluded.

The primary outcomes were the rates of penetrating trauma, suicide attempts, and drug and alcohol positivity. Urine drug toxicology and serum alcohol testing were not standardized across centers, however most of the participating centers perform routine screening for all trauma patients. Secondary outcomes included intensive care unit (ICU) admission, ICU length of stay (LOS), overall LOS, ventilator days, operations performed, and mortality. Vital signs upon arrival as well as demographic and injury data were collected including age, race, sex (self-reported), body mass index (BMI), insurance status (ie, private, uninsured, and Medicaid), and injury severity score (ISS). Mechanisms of injury were also recorded, including motor vehicle collisions (MVC), motorcycle collision (MCC), ground level falls, pedestrian struck, and assault.

Patients were divided into 3 groups based on injury date: a historical control from March 19, 2019, to June 30, 2019 (CONTROL), before SAH from January 1, 2020, to March 18, 2020, (PRE), and after SAH from March 19, 2020, to June 30, 2020 (POST). The POST group was compared to both the PRE and CONTROL groups in two separate analyses.

For all variables within each group, descriptive statistics were performed. Continuous variables were reported as means with standard deviation and categorical variables were reported as percentages. Either a two-sample *t*-test or Mann-Whitney *U* test was used to compare continuous variables and chi-square tests were used to compare categorical variables. A *P* value was considered significant if <.05. Data analyses were performed on IBM SPSS Statistics for Windows (version 24; IBM Corp., Armonk, NY).

## Results

A total of 726 adolescent trauma patients were included across the 3 time periods: 282 in the CONTROL group, 203 in the PRE group, and 241 in the POST group.

### Demographics

The 3 cohorts were similar in terms of age, sex, race, mean ISS and vital signs on arrival (all *P* > .05). Notably, there was a higher rate of Medicaid insurance patients in the POST compared to PRE (50.2% vs 31.0%, *P* < .001) and CONTROL (50.2% vs 38.3%, *P* = .006). Additionally, there was a lower rate of patients with private insurance in the POST compared to PRE (41.1% vs 54.7%, *P* = .004) and CONTROL (41.1% vs 52.1%, *P* = .012) (Table 1)

**Table 1.** Demographics of Adolescent Trauma Patients Compared by Time Period.

	POST	PRE	PRE vs POST	Control	Control vs POST
Characteristic	(n = 241)	(n = 203)	*P*-value	(n = 282)	*P*-value
Male, n (%)	174 (72.2%)	129 (63.5%)	.051	197 (69.9%)	.557
Age, years, mean ± sd	15.5 ± 1.3	15.4 ± 1.4	.981	15.3 ± 1.4	.240
Race/Ethnicity, n (%)					
white	99 (41.1%)	84 (41.4%)	.949	106 (37.6%)	.415
Latino	102 (42.3%)	94 (46.3%)	.400	124 (44.0%)	.705
Black	18 (7.5%)	7 (3.4%)	.067	17 (6.0%)	.511
Asian	7 (2.9%)	8 (3.9%)	.547	9 (3.2%)	.849
Insurance status, n (%)					
Medicaid	121 (50.2%)	63 (31.0%)	**<.001**^1^	108 (38.3%)	**.006**^1^
Private	99 (41.1%)	111 (54.7%)	**.004**^1^	147 (52.1%)	**.012**^1^
Uninsured	4 (1.7%)	12 (5.9%)	**.017**^1^	18 (6.4%)	**.007**^1^
Smoking	7 (2.9%)	6 (3.0%)	.975	5 (1.8%)	.389
BMI, kg/m2, mean ± sd	24.9 ± 6.0	23.6 ± 5.8	**.021**^1^	23.1 ± 5.2	**.001**^1^

sd = standard deviation, BMI = body mass index.

CONTROL = 3/19/19-6/30/19.

PRE = 1/1/20-3/18/20.

POST = 3/19/20-6/30/20.

**Bolded** values are significantly different.

^1^= significantly different in both comparisons.

### Injury Profile

The most common mechanism of injury across all time periods was MVC, with an incidence of 25.9%. The POST group had a similar penetrating trauma rate compared to both the PRE group (15.8% vs 13.8%, *P* = .56) and CONTROL group (15.8% vs 14.5%, *P* = .69), respectively. The POST group also had a similar rate of suicide attempt compared to both the PRE group (1.2% vs 1.5%, *P* = .83) and the CONTROL group (1.2% vs 2.1%, *P* = .43), respectively (Table 2).

**Table 2.** Injury Characteristics, Toxicology, and Vital Signs of Adolescent Trauma Patients Compared by Time Period.

	POST	PRE	PRE vs POST	Control	Control vs POST
Characteristic	(n = 241)	(n = 203)	*P*-value	(n = 282)	*P*-value
Mechanism of injury, n (%)					
Blunt	203 (84.2%)	175 (86.2%)	.560	241 (85.5%)	.696
Ground level fall	19 (7.9%)	19 (9.4%)	.580	27 (9.6%)	.496
Fall from height	15 (6.2%)	11 (5.4%)	.719	17 (6.0%)	.926
Pedestrian struck	19 (7.9%)	27 (13.3%)	.062	34 (12.1%)	.115
Motorcycle collision	14 (5.8%)	14 (6.9%)	.639	37 (13.1%)	**.005**
Motor vehicle collision	63 (26.1%)	59 (29.1%)	.492	66 (22.3%)	.311
Assault	12 (5.0%)	8 (3.9%)	.599	21 (7.4%)	.247
Sports injury	31 (12.9%)	27 (13.3%)	.892	36 (12.8%)	.974
Penetrating	38 (15.8%)	28 (13.8%)	.560	41 (14.5%)	.696
Gunshot	20 (8.3%)	18 (8.9%)	.831	22 (7.8%)	.835
Stab wound	13 (5.4%)	7 (3.4%)	.325	13 (4.6%)	.681
Suicide attempt, n (%)	3 (1.2%)	3 (1.5%)	.832	6 (2.1%)	.439
ISS, mean ± sd	7.8 ± 7.8	8.2 ± 8.3	.798	7.8 ± 9.4	.344
Alcohol positive, n (%)	48 (19.9%)	42 (20.7%)	.840	55 (19.5%)	.906
Urine toxicology positive, n (%)	69 (28.6%)	48 (23.6%)	.235	58 (20.6%)	**.032**
Vitals on arrival, mean ± sd					
Systolic blood pressure	129.7 ± 17.9	129.2 ± 16.9	.814	127.5 ± 18.0	.171
Respiratory rate	18.9 ± 4.3	19.7 ± 4.8	.051	18.4 ± 3.6	.467
Heart rate	97.7 ± 21.4	95.4 ± 18.9	.344	94.8 ± 21.8	.063
GCS score	14.0 ± 2.9	14.0 ± 2.7	.373	14.1 ± 2.7	.692

ISS = injury severity score, sd = standard deviation, GCS = glasgow coma scale

CONTROL = 3/19/19-6/30/19.

PRE = 1/1/20-3/18/20.

POST = 3/19/20-6/30/20.

**Bolded** values are significantly different.

### Drug and Alcohol Positivity

The POST group was similar in alcohol positivity to both the PRE (19.9% vs 20.7%, *P* = .84) and CONTROL group (19.9% vs 19.5%, *P* = .91). The POST group had increased overall drug positivity compared to the CONTROL group (28.6% vs 20.6%, *P* = .032), but not statistically significant compared to the PRE group (28.6% vs 23.6%, *P* = .24) (Table 2). The rates of individual drug use were not statistically different between any of the groups (all *P* > .05) (Table 3).

**Table 3.** Urine Toxicology Results of Adolescent Trauma Patients Compared by Time Period.

	POST	PRE	PRE vs POST	Control	Control vs POST
Characteristic	(n = 241)	(n = 203)	*P*-value	(n = 282)	*P*-value
Amphetamines	8 (3.3%)	5 (2.5%)	.594	4 (1.4%)	.148
Barbiturates	0 (.0%)	1 (.5%)	.275	0 (.0%)	n/a
Benzodiazepines	9 (3.7%)	13 (6.4%)	.197	7 (2.5%)	.407
Opioids	23 (9.5%)	8 (3.9%)	**.021**	21 (7.4%)	.389
Cocaine	5 (2.1%)	2 (1.0%)	.359	1 (.4%)	.066
PCP	0 (.0%)	0 (.0%)	n/a	1 (.4%)	.355
THC	49 (20.3%)	40 (19.7%)	.869	41 (14.5%)	.080
MDMA	0 (.0%)	0 (.0%)	n/a	0 (.0%)	n/a

PCP = phencyclidine, THC = tetrahydrocannabinol, MDMA =3,4-methyl​enedioxy​methamphetamine.

CONTROL = 3/19/19-6/30/19.

PRE = 1/1/20-3/18/20.

POST = 3/19/20-6/30/20.

**Bolded** values are significantly different.

### Secondary Outcomes

The POST group was similar to both groups in terms of ICU admission, ICU LOS, ventilator days, operations, and mortality (all *P* > .05). However, overall LOS was shorter in the POST group compared to the PRE group (2.5 ± 2.9 vs 3.6 ± 6.1, *P* = .032), but not statistically significant compared to the CONTROL group (2.5 ± 2.9 vs 3.6 ± 7.6, *P* = .12) (Table 4).

**Table 4.** Outcomes of Adolescent Trauma Patients Compared by Time Period.

	POST	PRE	PRE vs POST	CONTROL	CONTROL vs POST
Outcome	(n = 241)	(n = 203)	*P*-value	(n = 282)	*P*-value
LOS, days, mean ± sd	2.5 ± 2.9	3.6 ± 6.1	**.032**	3.6 ± 7.6	.128
ICU admission, n (%)	56 (23.2%)	62 (30.5%)	.083	56 (19.9%)	.348
ICU LOS, days, mean ± sd	.7 ± 1.8	1.5 ± 4.9	.061	1.1 ± 4.1	.466
Mechanical ventilation, n (%)	26 (10.8%)	25 (12.3%)	.615	20 (7.1%)	.137
Ventilator, days, mean ± sd	.3 ± 1.4	.8 ± 3.6	.554	.5 ± 2.8	.173
Operations, n (%)					
Tracheostomy	3 (1.2%)	3 (1.5%)	.832	3 (1.1%)	.846
Laparotomy	6 (2.5%)	9 (4.4%)	.259	11 (3.9%)	.364
Craniectomy/craniotomy	3 (1.2%)	5 (2.5%)	.336	8 (2.8%)	.206
Vascular/endovascular	1 (.4%)	2 (1.0%)	.465	1 (.4%)	.911
Discharge disposition, n (%)					
Home	195 (80.9%)	149 (73.4%)	.059	223 (79.1%)	.602
Long-term acute care hospital	2 (.8%)	3 (1.5%)	.519	3 (1.1%)	.784
Acute rehabilitation	2 (1.8%)	10 (4.9%)	**.008**	8 (2.8%)	.095
Mortality, n (%)	7 (2.9%)	5 (2.5%)	.775	8 (2.8%)	.963

LOS = length of stay, ICU = intensive care unit, sd = standard deviation.

CONTROL = 3/19/19-6/30/19

PRE = 1/1/20-3/18/20

POST = 3/19/20-6/30/20

**Bolded** values are significantly different

## Discussion

COVID-19 and the subsequent SAH orders have significantly changed both the adult and pediatric trauma populations.^7,13,14^ Unexplored to this point, this study examined the adolescent trauma population during California SAH orders. This retrospective multicenter study across Southern California found a similar rate of penetrating trauma, suicide attempts, and alcohol positivity in the adolescent trauma population after SAH orders. Interestingly, adolescents had increased drug positivity compared to a historical control, but not immediately prior to SAH orders. Furthermore, adolescents with Medicaid insurance comprised a larger proportion of traumatic injury after SAH orders compared to both immediately prior to SAH orders and a historical control.

Penetrating trauma rates, a surrogate for the level of violence within a population, has seen a notable rise after COVID-19 related SAH orders in adults.^15^ However, this study did not find a significant increase in penetrating trauma after SAH orders amongst adolescent trauma patients. For adolescents, risky behavior, such as engaging in violence, has been linked to social reward and peer influence.^16^ During SAH orders when many schools were moved to virtual platforms and large group gatherings were not allowed, adolescents likely spent less time with peers. This separation from peer social constructs may explain why penetrating trauma did not increase during SAH orders, as this population was exposed to less peer pressure to engage in violent behavior. Furthermore, adolescents may have had more parental supervision due to adults more commonly working from home, having fewer work hours, or being laid off during SAH orders.^17^ Additional research is needed to confirm these findings and if demonstrated may provide some framework for future intervention programs to mitigate adolescent firearm violence.

Substance abuse is common in adolescent patients in the United States, as an estimated 17.2% of this population has used illicit drugs in the past year.^18^ This current study demonstrated that urine toxicology positivity in adolescent trauma patients increased immediately after SAH orders when compared to a historical control. While there was an overall increase in drug positivity, we did not identify any statistically significant increase in any specific drug, although this may be due to a lack of statistical power. A possible increase in cocaine and THC use was noted and could be confirmed in a further study with a larger sample size. Regardless, the overall rise in drug use may be attributed to the increased stressors of the COVID-19 pandemic and SAH orders. Adolescents, a population already in a dynamic state of psychological and emotional growth,^11^ were exposed to social isolation^8^ potentially leading to drug use as an “escape” or attempted coping mechanism. This highlights the need for continued drug prevention efforts in this at-risk population, even during the current and/or any future pandemic.

Health inequities in medicine have received additional attention in recent years. A recent study examined the socioeconomic disparities in social distancing during the COVID-19 pandemic and showed that there was less social distancing in United States counties with higher numbers of essential workers and those below the poverty line.^19^ This current study demonstrates an increased rate of adolescent trauma patients with Medicaid after SAH orders. Similar findings have been described in the adult trauma population as well.^20^ This indicates that the COVID-19 SAH orders inadequately protected lower income individuals, possibly for the adult population because they were more likely to be part of the essential workforce and unable to work from home, and thus more likely to experience trauma. While the Medicaid adolescent population may not have a similar work burden, they may have had less adult supervision as their parents continued to work. These inequities deserve further exploration during the continuing pandemic.

This study has many limitations including those inherent to its retrospective design such as misclassification and missing data. Also, due to its post hoc design, no formal power analysis was performed and thus this study may be underpowered in identifying small but significant changes. Our collection period for this study also only extended a few months into the pandemic. In addition, significant missing pertinent variables include more detailed social and developmental history and pre-existing mental health diagnoses, which are important risk factors for adolescent trauma. Also, while the study incorporated 11 trauma centers, there was notably an absence of any free-standing children’s hospitals from the region. In addition, this study was conducted solely in Southern California which is a unique socioeconomic and geographical region and thus the results may not be generalizable to other regions across the United States or other regions of the world.

## Conclusion

This retrospective multicenter study demonstrated that adolescent trauma patients were affected differently by SAH orders than previously described for adults and children. Notably, adolescent trauma patients sustained a similar rate of penetrating trauma, suicide attempts, and alcohol positivity after SAH orders. Interestingly, adolescent trauma patients had increased drug positivity compared to the year prior. Finally, patients presenting during SAH orders more commonly had Medicaid insurance compared to the prior time period and a historical control group. These findings highlight the need for continued drug and injury prevention during a pandemic, as well as a focus on adolescent health disparities moving forward.
